# Dermatitis Herpetiformis as the Initial Presentation of Primary Biliary Cholangitis in a Male with Gluten Sensitivity

**DOI:** 10.7759/cureus.1247

**Published:** 2017-05-14

**Authors:** Cyriac Philips, Rajaguru Paramaguru, Divya A Indiran, Philip Augustine

**Affiliations:** 1 Hepatology and Liver Transplant Medicine, PVS Memorial Hospital; 2 Pathology, PVS Memorial Hospital; 3 Dermatology, Welcare Hospital, Kochi, Kerala, India; 4 Gastroenterology, PVS Institute of Digestive Diseases, PVS Memorial Hospital

**Keywords:** celiac disease, gluten sensitivity, dermatitis herpetiformis, cirrhosis, pbc, cholestasis, pruritus, portal hypertension, variceal bleeding, pathology

## Abstract

Celiac disease is commonly associated with elevated liver enzymes that normalize on a gluten-free diet. Celiac disease is rarely described in patients with primary biliary cholangitis. Dermatitis herpetiformis is the skin manifestation of the celiac disease that is very rarely associated with primary biliary cirrhosis. We present the case of a 62-year-old man who presented with severe chronic pruritus, in whom a diagnosis of primary biliary cholangitis was made initially. However, in the presence of atypical skin lesions, not confirming to chronic cholestasis, an in-depth evaluation including histopathological examination led to the diagnosis of dermatitis herpetiformis associated with gluten sensitivity. Gluten-free diet and medical treatment with dapsone led to beneficial clinical outcomes.

## Introduction

Dermatitis herpetiformis is a chronic recurrent epidermal disease most commonly associated with celiac disease and gluten hypersensitivity. Celiac disease is a systemic inflammatory disease secondary to gluten sensitivity in the presence of antibodies to tissue transglutaminase and is rarely associated with liver disease such as primary biliary cholangitis. The classical celiac disease association with liver disease is that of abnormal transaminases that normalize on a gluten-free diet [1]. We present the case of a 62-year-old man with primary biliary cholangitis and atypical skin lesions diagnosed as a case of dermatitis herpetiformis associated with gluten sensitivity, a rare association. Gluten-free diet and dapsone treatment led to clinical improvement.

## Case presentation

A 62-year-old man, teetotaler, diagnosed with insulin dependent diabetes mellitus for eight years without adequate glycemic control, complained of pruritus predominantly over the lower limbs, soles, palms, and upper arms for a period of two years that was worsening since past eight months with development of nodular erythematous skin lesions over sites of intense pruritus. Six months back he underwent endoscopic band ligation for acute variceal bleed, after that controlled on secondary prophylaxis with beta-blocker therapy. At the time, he was told to have cirrhosis of the liver, secondary to non-alcoholic fatty liver disease with skin changes suggestive of diabetic dermopathy and was advised strict glycemic control, emollients, and anti-histamines. After a period of initial improvement, the pruritus gradually worsened, and new crops of lesions developed over the lower back and thighs with new onset fatigue developing over the past two months. He visited our outpatient department after that. On physical examination, only mild bilateral pedal pitting edema was evident. However, multiple variable-sized erythematous nodular lesions, showing central scaling, bleeding spots or hyperpigmentation, and several excoriations were present over the thighs, legs, upper arms and lower back, some of them showing confluence. Serologies for hepatitis A, B, C, and E were negative, and HbA1c was 6.8%. Vitamin B-12, serum folic acid levels, and iron studies were within normal limits. Blood investigations for antibodies to nuclear, liver-kidney-microsomal type one, soluble liver, smooth muscle, Smith and double-stranded deoxyribonucleic acid were negative and total serum immunoglobulin G levels were within normal limits. However, the anti-mitochondrial antibody was positive at 1:80 titer by enzyme immunoassay test. Relevant blood investigations are shown in Table 1.

**Table 1:** Blood investigations Relevant past and current blood investigations of the patient.

Parameter	Patient result (initial evaluation elsewhere)	Patient result (current admission)	Upper limit of normal
Hemoglobin (g/dL)	10.2	10.0	16
Total leucocyte count (per litre)	4200	3800	10,000
Platelet count (per litre)	120,000	90,000	450,000
Total bilirubin (mg/dl)	2.4	3.1	1.0
Direct bilirubin (mg/dl)	1.2	1.8	0.2
Aspartate transaminase (IU/L)	34	42	42
Alanine transaminase (IU/L)	46	48	41
Alkaline phosphatase (IU/L)	308	688	132
Gamma glutamyl trans peptidase (IU/L)	299	267	82
Serum albumin (g/dL)	3.2	3.1	5.0
Total cholesterol (mg/dl)	-	288	200
Triglycerides (mg/dl)	-	304	150
High density lipoprotein (mg/dl)	-	48	60
Low density lipoprotein (mg/dl)	-	142	130

**Figure 1:**
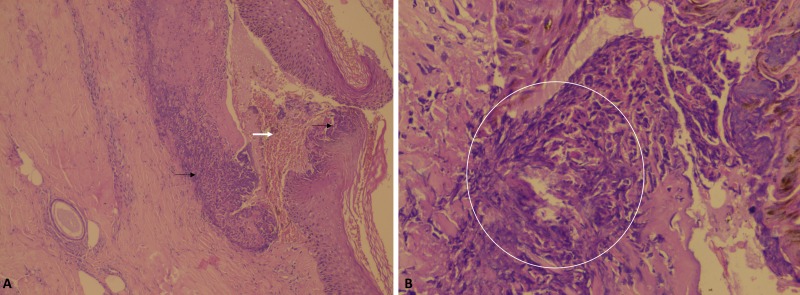
Skin histopathology Skin histopathology showing hyperplastic stratified squamous epithelium (A, black arrows) with focal epidermal papule formation (A, white arrow) with focal sub-epidermal dense neutrophilic infiltrates (B, circled area) suggestive of microabscesses with papillary dermal necrosis.

Abdominal ultrasound, computer tomography, and magnetic resonance cholangiopancreatogram scans showed the cirrhotic architecture of the liver with splenomegaly without ascites, focal liver lesions, portal vein thrombosis, intrahepatic biliary radicle dilatation and normal common bile and pancreatic ducts. Bone densitometry, also called dual-energy x-ray absorptiometry or DEXA was suggestive of osteopenia. In view of the chronic cholestatic pattern of liver function tests, presence of anti-mitochondrial antibodies and vitamin D deficiency, a diagnosis of primary biliary cholangitis or PBC was made as the cause of liver disease. Ursodeoxycholic acid at 13 mg/kg per day in divided doses was started. However, the skin lesions were not classical of PBC. Skin biopsies were taken from inactive and active lesions, the histopathology revealing hyperplastic stratified squamous epithelium with hyperkeratosis, hypogranulosis, mild perivascular lymphoplasmacytic and neutrophilic dermal infiltrates in the former and hyperplastic epidermis with focal epidermal papule formation with focal sub-epidermal dense neutrophilic infiltrates suggestive of microabscesses with papillary dermal necrosis was noted in the latter (Figure 1).

**Figure 2:**
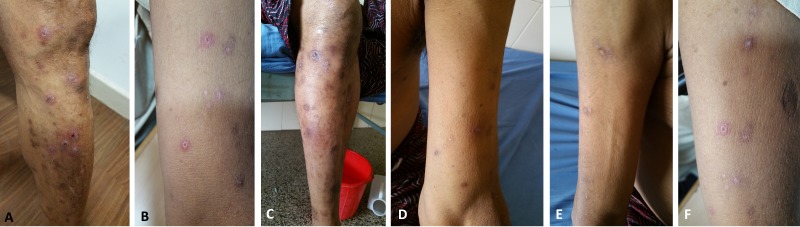
Clinical course of skin lesions Initial skin examination was showing multiple variable sized erythematous nodular lesions, showing central scaling, bleeding spots or hyperpigmentation and several excoriations present over the legs and upper arms with some of them showing confluence (A and B). On a gluten-free diet and dapsone medication, the activity of the lesions subsides, and most lesions fade away (C, D, and E) only to come back on rechallenge with dietary gluten (F).

These findings were suggestive of dermatitis herpetiformis and its differentials. To confirm the diagnosis, we performed a repeat skin biopsy encompassing normal and abnormal areas and direct immunofluorescence test for immunoglobulin A deposits. However, this was noncontributory. Immunoglobulin A antibody to tissue transglutaminase was strongly positive at 202 U/mL, the upper limit of normal, less than 10. Considering celiac disease as an association, an upper gastrointestinal endoscopy with multiple deep duodenal biopsies was performed, which did not show significant pathology. To characterize the clinical course further and to confirm the diagnosis of dermatitis herpetiformis, the patient was advised gluten-free diet and 100 milligrams of oral dapsone was added. In three days’ time, the skin lesions started showing resolution in activity and pruritus reduced substantially. Dapsone was stopped and dietary gluten was restarted. The patient developed severe itching with new active lesions after four days of rechallenge, confirming the diagnosis of dermatitis herpetiformis and gluten sensitivity (Figure 2).

After that, he was advised to continue ursodeoxycholic acid and the low dose of naltrexone with fat soluble vitamin replacement and the short course of dapsone for symptomatic relief. At one month follow-up, the patient is keeping well and with abatement in symptoms.

## Discussion

Primary biliary cholangitis is an autoimmune liver disease with a female preponderance, affecting those aged between 40 and 60 years and characterized by progressive destruction of the intra-hepatic and inter-lobular bile ducts leading to cholestasis, fibrosis, and cirrhosis in the long term. The diagnosis of PBC can be established when two of the following three criteria are met – biochemical evidence of cholestasis based mainly on alkaline phosphatase elevation, the presence of antimitochondrial antibody and histologic evidence of non-suppurative destructive cholangitis and destruction of interlobular ducts. The antimitochondrial antibody is a highly disease-specific autoantibody found in up to 95% of patients with PBC [1-2]. Fatigue is the most common clinical manifestation of PBC and is present in up to 80% of patients. Pruritus is the second most common symptom in PBC and affects 40 to 80% of patients with hyperlipidemia and metabolic bone disease affecting around 75 to 80% of patients with PBC. Chronic pruritus, defined as itch lasting for more than six weeks due to underlying liver disease, is most commonly associated with cholestasis, typically seen with PBC or primary sclerosing cholangitis and is not classically associated with nodular or chronic eruptive skin lesions, is generalized, also affecting the palms and soles, which was lacking in our patient. Other conditions that can cause severe chronic pruritus include scabies, aquagenic pruritus of polycythemia, paraneoplastic pruritus syndromes such as granuloma annulare, dermatomyositis, transient acantholytic dermatosis or Grover's disease, erythroderma, pemphigus, eruptive seborrheic keratosis with pruritus or Lesser Trelat sign, malignant acanthosis nigricans and Bazex syndrome or acrokeratosis paraneoplastica and psychogenic itching; all of which were ruled out in our patient based on history, clinical evaluation, imaging, and histopathology [2]. Extrahepatic manifestations are common in patients with PBC and typically include Sjogren's syndrome, Hashimoto's thyroiditis or Grave's disease, hypothyroidism, systemic sclerosis and less commonly, rheumatoid arthritis, systemic lupus erythematosus and celiac disease [3].

Celiac disease or CD is a systemic autoimmune disorder, caused by the interplay between tissue transglutaminase identified as the major autoantigen, and gliadin, which is contained in wheat, oats, rye and barley and is a common disorder with a prevalence of one percent in the general population with a higher frequency in women occurring only in genetically predisposed individuals sharing a particular haplotype, that is, human leukocyte antigen HLA-DQ2 and/or -DQ8. However, we did not perform HLA testing as it was not required for confirmation of CD diagnosis. The clinical picture is variable and characterized by various intestinal symptoms such as diarrhea, constipation, bloating and/, or abdominal pain and extra-intestinal symptoms or signs of fatigue, anemia, aphthous stomatitis, osteopenia or osteoporosis, recurrent miscarriages and elevated transaminases or can be asymptomatic. Diagnostic criteria include serology and small intestinal histopathology. Tissue transglutaminase antibodies and endomysial antibodies of the immunoglobulin A class show the highest diagnostic accuracy for CD and the recently identified deamidated gliadin peptide antibodies of immunoglobulin G class are valuable for diagnosis of CD in patients with IgA deficiency and children less than two years of age. Small bowel biopsy can show features ranging from flat mucosa to mild intestinal lesions and is still regarded as the diagnostic gold standard for CD in adults [4]. However, latent CD has been reported in adults with dermatitis herpetiformis, in which the architecture of the small intestine is usually normal. These patients can develop classical CD on follow-up or may even present with small bowel lymphoma. Dermatitis herpetiformis is called as CD of the skin [5]. Hence, in the absence of classical findings, we could not still rule out the evolution of CD in this patient and hence utilized the term, gluten sensitivity.

Dermatitis herpetiformis associated with CD and PBC is very rarely documented in the literature. Gabrielsen and Hoel reported the case of a 69-year-old man with a previous diagnosis of PBC presenting with a highly pruritic rash on the knees, elbows, and in the inguinal region which was both clinically and histologically compatible with dermatitis herpetiformis, confirmed with dapsone treatment [6]. Walton and Walton described a male patient with diabetes mellitus in whom PBC was diagnosed eight years after he presented with dermatitis herpetiformis and the authors, on subsequent review of 22 other patients with dermatitis herpetiformis, failed to reveal any patients with antimitochondrial antibodies or abnormal liver function tests [7]. Dermatitis herpetiformis, also known as Duhring-Brocq dermatitis, is a chronic, recurrent disease, secondary to gluten hypersensitivity characterized by the papulovesicular pruriginous rash. Celiac disease is the gastrointestinal manifestation of the same etiology; however, dermatitis herpetiformis patients rarely have gastrointestinal symptoms, but some, not all, present with varying degree of intestinal villous atrophy. This skin condition is characterized by the presence of immunoglobulin deposits on top of the dermal papillae and manifests itself mainly on the extensor surface of the limbs, buttocks, and scapular area. Patients have circulating levels of immunoglobulin A against transglutaminase with clinical remission on a gluten-free diet. The main differential diagnoses include bullous pemphigoid which presents with large tense blisters on flexor surfaces, trunk, intertriginous regions and mucosa without microabscesses formation and linear immunoglobulin A dermatosis in which homogenous band of immunoglobulin A at the dermal-epidermal junction is seen on direct immunofluorescence in the absence of anti-endomysial or anti-tissue transglutaminase antibodies and is not gluten sensitive. Through a clinical challenge test, we ruled out these two close possibilities in our patient [8]. We stopped dapsone and restarted gluten in the diet to confirm the diagnosis of dermatitis herpetiformis through a re-challenge. It has been shown that tapering of dapsone doses results in no flaring of disease in significant numbers of patients with dermatitis herpetiformis and hence, the resurgence of symptoms in our patient could be attributed to gluten re-introduction [9].

Our patient's presentation is unique in many ways. The presentation of PBC and that of complications of portal hypertension was with dermatitis herpetiformis which was not associated with CD but was related to strong positivity towards antibodies to tissue transglutaminase and severe gluten sensitivity. In a study by Niveloni, et al., three patients out of 10 with PBC presented evidence of gluten sensitivity. All three had abnormal titers of an antigliadin antibody of immunoglobulin A, and one was positive for the endomysial antibody. One of them also exhibited other features of gluten sensitivity, but despite evidence of gluten intolerance, patients had minimal or no symptoms characteristic of celiac disease [10]. In our patient, in the absence of a classical diagnosis of CD, PBC was still associated with dermatitis herpetiformis and antibodies to tissue transglutaminase with improvement on gluten-free diet and classical relapse on rechallenge with gluten diet; an association which is rare and novel.

## Conclusions

Primary biliary cholangitis has a multitude of extrahepatic disease associations, the knowledge of which are important in clinical decision making and treatment. In a cirrhotic presenting with chronic pruritus, looking beyond classical dermatological manifestations of cholestatic liver disease is of great value in identifying rare, but treatable causes of associated conditions. The association of CD with PBC is well known, but presentation with dermatitis herpetiformis in the absence of CD is very rare. Screening for PBC in patients with CD and vice versa is advocated. In the presence of atypical symptoms and unusual clinical association, histopathology evaluation has a beneficial role.
